# Perceptions and Knowledge of Nuts amongst Health Professionals in New Zealand

**DOI:** 10.3390/nu9030220

**Published:** 2017-03-01

**Authors:** Rachel C. Brown, Lee Ching Yong, Andrew R. Gray, Siew Ling Tey, Alexandra Chisholm, Sook Ling Leong

**Affiliations:** 1 Department of Human Nutrition, University of Otago, P.O. Box 56, Dunedin 9054, New Zealand; yonle133@student.otago.ac.nz (L.C.Y.); siewling.tey@otago.ac.nz (S.L.T.); alex.chisholm@otago.ac.nz (A.C.); 2 Nutrition Society of New Zealand, Whanganui 4543, New Zealand; 3 Department of Preventive and Social Medicine, University of Otago, P.O. Box 56, Dunedin 9054, New Zealand; andrew.gray@otago.ac.nz; 4 Department of Surgical Sciences, University of Otago, P.O. Box 56, Dunedin 9054, New Zealand; sookling.leong@otago.ac.nz

**Keywords:** nuts, consumption, health professionals, knowledge, perceptions, cardioprotective

## Abstract

Despite their nutritional value, population-level nut consumption remains low. Studies suggest that individuals would eat more nuts on their doctor’s advice, making health professionals potentially important for promoting nut consumption. This cross-sectional study aimed to examine the perceptions and knowledge of nuts and the predictors of nut promotion among health professionals in New Zealand. Dietitians, general practitioners (GPs), and practice nurses were identified from the Electoral Roll and invited to complete a questionnaire (*n* = 318, 292, and 149 respondents respectively). Over one-fifth of GPs and practice nurses believed that eating nuts could increase blood cholesterol concentrations and cause weight gain. The most common perceptions overall were that nuts are healthy; high in protein, fat, and calories; and are satiating. Nut consumption was recommended for reasons relating to these perceptions and because of nuts’ selenium content. Conversely, reasons for suggesting the consumption of fewer nuts included that they were high in calories and fat, would cause weight gain, and concerns regarding allergies and cost. All groups of health professionals were more likely to promote nut consumption if they perceived nuts to reduce the risk of diabetes (all *p* ≤ 0.034). Education could improve health professionals’ knowledge regarding the effects of nut consumption on blood cholesterol and body weight, alongside other health benefits, which should improve the advice given to patients and may thereby increase nut consumption.

## 1. Introduction

Nuts are rich in *cis*-unsaturated fats, fibre, vitamins, minerals, and a number of phytonutrients [1,2,3]. The regular consumption of nuts has been shown to be associated with a reduction in total mortality [4,5], with a reduction in the risk of cardiovascular disease (CVD) appearing to be the main driver of this alongside reduced morbidity [3,6,7,8]. Table 1 summarises the evidence for the health effects of nut consumption.

The National Heart Foundation of NZ recommends the consumption of 30 g of nuts per day as part of a cardioprotective diet [9]. Similarly, in the United States of America (USA), a qualified health claim stating that eating 42 g of nut per day may reduce the risk of heart disease was approved in 2003 [10]. However, information from population studies indicates that regular nut consumption is far below these guidelines [11,12,13,14]. For example, a nationally-representative survey in NZ showed that only 29% of New Zealanders consumed any form of nut on the surveyed day, with a mean intake on that day of 18 g among these nut consumers, resulting in a mean intake of only 5 g across the whole population [11]. Furthermore, only 6.9% of respondents consumed whole nuts that day, with a mean population intake of only 3 g/day, although those consuming whole nuts on the surveyed day had a mean consumption of 40 g, above the NZ and around the USA recommended daily intakes. Similar data has been reported in Europe and the USA, where population-level rates for whole nut consumption were 6.9% and 6.0%, respectively [12,13].

Given these low levels of nut consumption relative to the guidelines, it is important to examine the potential facilitators of, as well as barriers to, regular nut consumption. One study from the USA, which examined the perceived benefits of and barriers to nut consumption among low-income people, reported that participants strongly agreed with the statement that they would eat more nuts if their doctor recommended them to do so [15]. In a further study by Pawlak et al., among those with or at high risk of CVD and/or diabetes, only 27% of respondents reported that their doctor advises them to eat nuts. However, 64% agreed that they would consume nuts on most days of the week if their doctor provided such a recommendation [16]. Therefore, there appears to be a potentially important role for health professionals in encouraging regular nut consumption as a means of reducing chronic disease risk. To the best of our knowledge, however, no studies have investigated how nuts are perceived by doctors, or indeed any health professional groups, and how these perceptions influence their promotion of nuts. Similarly, we are not aware of any studies that examine the knowledge of nuts and associations between nut consumption and health amongst health professionals. Given that advice from health professionals may be an important facilitator of nut consumption among the general population, this information is important. Therefore this study aimed to assess the perceptions of and knowledge about nuts and predictors of nut promotion and demotion among health professionals, including dietitians, general practitioners, and practice nurses.

## 2. Materials and Methods

### 2.1. Study Design and Participants

This was a cross-sectional study of health professionals identified from the NZ Electoral Roll (as extracted on the 25 of July 2014) and was conducted during September–November 2014. All NZ citizens and permanent residents 18 years or older are required by law to enrol to be registered on the Parliamentary Electoral Roll, and the roll is estimated to include 92.6% of all adults [41]. Self-described occupations are included on this roll. After exploring this information to identify likely variants and to exclude descriptions that were likely to include those ineligible to participate (e.g., simply ‘doctor’ or ‘nurse’), we identified individuals whose occupation fell under three main categories, dietitians, general practitioners (GPs), and practice nurses, using the following occupation descriptions (including spelling variants):

1. *Dietitians*: ‘Dietitian’, ‘Clinical Dietitian’, ‘Clinical Dietician’, ‘Public Health Dietician’, ‘Sports Dietician’, or ‘Registered Dietitian’;
2. *General Practitioners (GPs)*: ‘General Practitioner’, ‘GP’, ‘General Practitioner Doctor’, ‘Family Doctor’, ‘Medical General Practitioner’, ‘General Medical Practitioner’, ‘Family Practitioner’, or ‘Family Physician’;
3. *Practice nurses*: ‘Practice Nurse’, ‘Nurse Practitioner’, ‘Community Nurse’, ‘Community Nursing’, or ‘Community Health Nurse’.

These professions reflect those who we considered the most likely to provide dietary advice. A dietitian is a registered health professional who helps to promote nutritional well being, treat disease, and prevent nutrition-related problems. A GP (family doctor) is a medical doctor who works in primary care to treat acute and chronic illnesses, including making referrals to secondary care, and provides preventive care and health education to patients. Practice nurses work alongside a GP in a health clinic and are generally involved in health promotion and education.

An information sheet was sent to all participants, and completion of the survey was taken as informed consent. The study was approved by the University of Otago Ethics Committee (reference number D14/288).

### 2.2. Survey Development

The self-administered questionnaire included questions on the following socio-demographic characteristics; age, sex, ethnicity (including multiple ethnicities), and number of years working as a registered practitioner. The questionnaire assessed respondents’ perceptions and knowledge of nuts (which included nut butters and peanuts but not coconuts or chestnuts, as these differ in composition), reasons for recommending patients to eat more or fewer nuts (including nut butters), and the types and forms of nuts that were recommended. The questionnaire was developed by the authors, who include a registered dietitian (AC), before being pre-tested among a group of twelve health professionals, including six dietitians, two general practitioners, and four practice nurses, and modified where appropriate, establishing both face and content validity. Both an online version and a paper version of the questionnaire were produced in order to optimise the response rates through providing respondents with both modalities.

### 2.3. Survey Administration

Recruitment followed an adaptation of Dillman’s four stage tailored method for mail surveys [42]. Firstly, the selected health professionals were mailed an invitation to participate, which included a cover letter and an information sheet regarding the study. The cover letter contained the web address for the online questionnaire. Secondly, seven days after the first mail out, all health professionals in the sample were sent a postcard, which served as a thank-you to those who had completed the questionnaire and a reminder to those who had not. Thirdly, 15 days after the initial mail out, a second invitation with a paper version of the questionnaire was sent to the non-respondents. Finally, 27 days after the initial mail out a second postcard was sent to all recipients of the third mail-out, which again served as a thank-you to those who had since completed the questionnaire and a reminder to those who had not. Users were provided with a login code so that each participant could only complete the questionnaire once.

### 2.4. Statistical Analysis

Initially, in order to have 95% confidence interval half-widths of 7.5% (worst case) for the proportions within each occupational group, 184 usable responses were required, equivalent to 378 (rounded to 400) potential respondents in each category, allowing for response rates of approximately 50%. After extracting the identified occupations from the Electoral Roll, it was apparent that sufficient numbers were not available for all three categories and all identified electors were included in the sample for a total of 1440 health professionals, comprising 578 dietitians, 596 general practitioners, and 266 practice nurses, giving 95% confidence interval half-widths, assuming 50% participation, of 6.0%, 5.9%, and 8.9% respectively, with the greater numbers of dietitians and general practitioners providing approximately the same power as the original numbers for comparisons between either of these larger groups and the smaller number of practice nurses.

The baseline characteristics of respondents are presented as arithmetic means and standard deviations for continuous variables. Categorical data are presented as frequencies and percentages. Self-reported ethnicity was categorised based on a priority classification system using Statistics New Zealand’s level one groups and a coding prioritisation order (from highest to lowest) of Māori, Pacific, Asian, Middle Eastern/Latin American/African (MELAA), other, and European [43]. For example, if a participant identified as both Māori and European, they were classified as having a prioritised ethnicity of Māori. For analysis purposes, to ensure sufficient numbers in each group, these were combined into four groups as follows: Māori, European, Asian, and other (comprising the Pacific, MELAA, other, and residual categories in Statistics New Zealand’s level one ethnicity categories) [43]. Characteristics were compared between health professions using a Chi-squared test for sex, a one way ANOVA for age, Fisher’s Exact test for ethnicity, and Kruskal-Wallis for years as a registered practitioner.

For categorical outcome variables, unadjusted differences in binary variables between health profession groups were examined using logistic regression when there were at least 20 respondents in both categories (using the guidelines from Peduzzi, et al. (1996) [44]); the Chi-square test when there were no more than 20% of expected cell frequencies below five but too few, following Peduzzi et al.’s guidelines; and Fisher’s Exact test otherwise. Where the number of responses was sufficient (i.e., ≥70 given the seven parameters being estimated), the logistic regression models were also adjusted for age (continuous), sex (male, female), and ethnicity (four levels), as these could potentially differ between occupations and be associated with responses. For all models including age as an independent variable, age-squared was assessed as an additional variable to identify non-linear associations. The Hosmer-Lemeshow test and a model specification test were used to assess goodness of fit. In a similar way, linear regression was used to compare perception scores between health professions on Likert-type items. Responses were coded as follows; strongly agree = 1, agree = 2, neither agree nor disagree = 3, disagree = 4, and strongly disagree = 5. For the linear regression models, outcome variables were log-transformed where this improved residual normality and/or homoscedasticity. Where this was not sufficient to satisfy model assumptions, quantile regression was used instead to compare medians. Where regression (linear, logistic, or quantile) models were used and the overall test for differences between health professionals was significant, pairwise comparisons between groups were made without further adjustment for multiple comparisons.

Stata Statistical Software 12.1 (Statacorp LP, College Station, TX, USA) was used for all statistical analyses. Missing responses were relatively infrequent for each question and no special treatment for these was performed. All statistical tests were two-sided, and *p <* 0.05 was considered statistically significant.

## 3. Results

### 3.1. Response Rate

In total, 759 of the 1440 (53%) health professionals completed the questionnaire, which was slightly higher than the anticipated response rate of 50%. The response rates by health profession were not significantly different, being 55% for dietitians, 49% for GPs, and 56% for practice nurses (Chi-squared *p* = 0.058), and no reasons were given by participants for not completing the questionnaire.

### 3.2. Participant Demographics

Dietitians were the largest professional group, with all groups differing significantly in terms of sex, age, ethnicity, and number of years’ experience (all *p* ≤ 0.002) (Table 2). Dietitians and practice nurses were predominantly women (both over 95%), with GP respondents closer to equality (57% women). The mean age was 47.3 years, with the dietitian group on average being around 8 years younger than both GPs and practice nurses. The respondents had a median of 20 years as registered practitioners, with practice nurses having being registered five and 12 years longer than GPs and dietitians, respectively. The majority of respondents were NZ European (86%), with more Asian and MELAA respondents in the GP group.

### 3.3. Perceptions and Knowledge about Nuts and Nut Butters among Health Professionals

Table 3 presents the perceptions and knowledge regarding nuts and nut butters among health professionals. On the whole, all three groups of health professionals mostly agreed that nuts are healthy, high in protein and fat, and filling and disagreed that they were low in calories (over three-quarters for each).

Among those who provided an actual response (i.e., did not answer ‘do not know’), there were significant differences in agreement between health professionals for several perceptions in the unadjusted model, which remained significant after adjustment for age, sex, and ethnicity. Dietitians were more likely to agree that nuts are healthy, high in fat, and high in selenium (for some nuts) and were more likely to disagree that nuts can increase the risk of cardiovascular disease (CVD), can increase blood cholesterol levels, are naturally high in sodium, are low in fibre, and are low in vitamins and minerals compared to both practice nurses and GPs (all pairwise *p* ≤ 0.044). Nurses were more likely to agree that nuts are high in iron and less likely to disagree that nuts are low in vitamins, minerals, and energy compared to both dietitians and GPs (both pairwise *p* ≤ 0.044). GPs were less likely to agree that nuts are high in antioxidants compared to dietitians (pairwise *p* = 0.011).

There were also overall differences between health professionals in terms of the percentage who responded ‘do not know’ to a number of perceptions/knowledge statements. Due to the low number of ‘do not know’ responses, only four outcomes could be further adjusted for age, sex, and ethnicity; therefore unadjusted pairwise comparisons are presented in the first instance, followed by a description of adjusted comparisons. Pairwise comparisons showed that, compared to dietitians, both GPs and nurses were more likely to answer ‘do not know’ for the statements that some nuts are high in selenium, nuts are naturally high in sodium, nuts are high in antioxidants and iron, and eating nuts can lower blood cholesterol (all pairwise *p* ≤ 0.014). In addition, GPs were more likely than dietitians, to respond ‘do not know’ for the perceptions that nuts are filling, are low in vitamins and minerals, are low in fibre, and lower the risk of diabetes (all pairwise *p* ≤ 0.014). A higher percentage of nurses responded ‘do not know’ to the statement that nuts are high in protein compared to dietitians (pairwise *p* = 0.032). After adjusting for age, sex, and ethnicity, the only change was for the statement that eating nuts can lower the risk of diabetes, wherein the overall difference between health professionals became a non-statistically significant tendency (*p* = 0.050).

### 3.4. Reasons for Advising Patients to Eat More Nuts and/or Nut Butter

Of the entire sample, 68% said they recommend that their patients consume more nuts. Table 4 shows the percentage of these who agreed with the listed reasons for advising patients to consume more nuts and/or nut butters. The most common reasons given across the entire sample for advising nut consumption was because they were perceived as healthy; good sources of protein, unsaturated fats, energy, and selenium; and promoting satiety. Less than two-thirds of health professionals advised eating more nuts to decrease the risk of CVD and less than one-half for helping lower blood cholesterol.

There were significant differences between health professionals for several of the reasons for promoting nut intake in the adjusted model. Significantly more dietitians promoted nuts because they are a good source of calories than did GPs or practice nurses (both pairwise *p* ≤ 0.007), while significantly fewer promoted nuts as a good source of iron compared to GPs and practice nurses (both pairwise *p* ≤ 0.046). In addition, compared to GPs, significantly more dietitians recommended nuts because they were a good source of unsaturated fat (pairwise *p* < 0.001), to reduce the risk of CVD (pairwise *p* = 0.013), for their cholesterol-lowering properties (pairwise *p* = 0.012), and as a good source of fibre (pairwise *p* = 0.003). More dietitians and GPs recommend eating nuts because they promote satiety, compared to practice nurses (both pairwise *p* ≤ 0.008).

### 3.5. Reasons for Advising Patients to Eat Fewer Nuts and Nut Butters

Table 5 shows the percentage of health professionals who agreed with the listed reasons for advising patients to consume fewer nuts and nut butters. The most common reasons across the entire sample were that nuts are high in calories and fat, that regular consumption would cause weight gain, concerns with nut allergies, and that nuts are considered too expensive for their patients, although only the first reason exceeded 50%.

Due to the limited number of variables that could be examined in adjusted models, the unadjusted differences are highlighted here. Among those questions with overall significant results, dietitians were less likely than both GPs and practice nurses to recommend that people eat fewer nuts because they are high in fat (both pairwise *p* ≤ 0.039), could increase blood cholesterol (both pairwise *p* ≤ 0.001), are naturally high in salt (both pairwise *p* ≤ 0.004), and can increase the risk of CVD (both pairwise *p* ≤ 0.002). Dietitians were also less likely than both other professions to recommend that people eat fewer nuts because they don’t know enough about nuts and their benefits (both pairwise *p* ≤ 0.028). Both dietitians and nurses were more likely than GPs to recommend the consumption of fewer nuts because of dental issues that make them inconvenient/uncomfortable to eat (both pairwise *p* ≤ 0.034). Practice nurses were significantly more likely than GPs to recommend eating fewer nuts because they are too expensive (pairwise *p* = 0.011). Furthermore, practice nurses were more likely than dietitians to recommend eating fewer nuts because there is conflicting information and they do not want to confuse patients (pairwise *p* = 0.019). Due to the low number of negative responses to these questions, only three reasons could be further adjusted for age, sex, and ethnicity. These were that nuts are healthy, high in fat, and regular consumption may increase body weight. There were no significant differences between health professionals for these three reasons after adjustment (all overall *p* ≥ 0.148) with the previously significant difference around fat perceptions becoming non-significant.

### 3.6. Perceptions of Nuts as Predictors of Nut Promotion among Health Professionals

We identified several perceptions of nuts that predict whether or not health professionals would be likely to recommend nuts (Table 6). Dietitians were more likely to recommend nuts in both the unadjusted and adjusted models if they perceived nuts to be healthy (both *p* < 0.001), as reducing the risk of diabetes (both *p* ≤ 0.017), and if they disagreed that they increase blood cholesterol (both *p* ≤ 0.013) or the risk of CVD (both *p* ≤ 0.018). Dietitians who perceived nuts to be high in selenium were more likely to recommend them in the unadjusted model (*p* = 0.049), but this became a non-statistically significant tendency after adjustment for age, sex, and ethnicity (*p* = 0.057).

GPs were more likely to recommend nut consumption in both the unadjusted and adjusted models if they perceived nuts to be healthy (both *p* < 0.001), filling (both *p* ≤ 0.016), high in selenium (both *p* ≤ 0.001), high in antioxidants (both *p* ≤ 0.023), high in iron (both *p* < 0.001), and able to lower the risk of diabetes (both *p* < 0.001) and if they disagreed that nuts are low in vitamins and minerals (both *p* < 0.001), low in fibre (both *p* < 0.043), naturally high in sodium (both *p* < 0.001) or that eating them would increase the risk of CVD (both *p* < 0.001), increase blood cholesterol levels (both *p* ≤ 0.005), or cause weight gain (both *p* ≤ 0.010).

In the unadjusted model, practice nurses were more likely to recommend nuts if they disagreed that eating nuts can increase the risk of CVD (both *p* < 0.039), nuts are naturally high in sodium (both *p* < 0.012), nuts are low in fibre (both *p* < 0.040), and eating them causes weight gain (both *p* < 0.031). In the fully adjusted model, general practice nurses were more likely to recommend nuts if they thought nuts would lower the risk of diabetes (both *p* = 0.034) and if they disagreed that nuts are naturally high in sodium (both *p* = 0.017) and low in fibre (both *p* = 0.042).

There was evidence that the way beliefs were associated with the odds of recommending nuts differed between the three professions for two questions, namely that nuts are healthy (overall interaction *p* = 0.042) and eating nuts can lower risk of diabetes (overall interaction *p* = 0.050), with weaker effects observed amongst practice nurses for both.

### 3.7. Perceived Healthiness of Peanuts Compared to Tree Nuts

As shown in Table 7, almost 50% of dietitians rated the healthiness of peanuts about the same as that of tree nuts, compared to only around one-quarter of GPs and practice nurses. The majority of GPs and practice nurses rated peanuts as ‘slightly less healthy’ than tree nuts, with around 20% rating them as ‘much less healthy’. From the quantile regression models, the median rating was significantly different between health professionals both in the adjusted and unadjusted models (both overall *p* ≤ 0.001), with significant differences between dietitians and both GPs and practice nurses (both pairwise *p* < 0.001). There was no evidence of a difference in median ratings between GP and practice nurses (pairwise *p* = 1.000 in unadjusted and adjusted models).

## 4. Discussion

To the best of our knowledge, this is the first study to examine beliefs about and perceptions of nuts using a sample drawn from different health professions, enabling the first comparisons between these groups. How health professionals perceive nuts is likely to influence the advice they offer to patients. Therefore, gaining an understanding of this is important, especially given that nut consumption in a number of countries is lower than recommended [11,12,13]. Also, previous research has suggested that individuals would consume more nuts if advised to do so by a doctor [15] and this would appear likely to also apply to other groups of health professionals. Dietitians and practice nurses also offer dietary advice, and examining these professionals is important alongside examining doctors. We identified gaps in health professionals’ knowledge, which could be used to develop educational material aimed at specific health professions, specifically targeting perceptions of nuts, that our results suggest could lead to an increased likelihood of health professionals recommending the consumption of nuts, which in turn could promote nut consumption. An evidence-based brochure regarding the health benefits of nuts, including special populations, could be developed to standardise advice and minimise the risk of confusion amongst both practitioners and patients. A breakdown of the properties of different nuts could also be included. Results of this study may be generalisable to health professionals in other countries with similar healthcare systems and dietary patterns to New Zealand and possibly more broadly with greater caution. The extent of training that non-dietitian health professionals in other countries receive in nutrition, in particular, could be expected to have an impact on between-profession results.

We found that all three health professions largely perceived nuts as healthy and high in calories, fat, protein, vitamins, and minerals, although agreement among the dietitian group was more pronounced than among GPs and practice nurses. These perceptions were similar to the reasons the health professionals provided for advising patients to eat more nuts.

There were some differences in the perceptions of nuts between health professionals. Significantly more dietitians agreed that some forms of nuts are high in selenium. This is likely due to the specialised nutrition knowledge of dietitians. However, overall two-thirds of health professionals agreed with this statement. The high level of knowledge on this topic among this group of NZ health professionals is likely due to the well-known low levels of selenium in NZ soils and the long history of low plasma selenium within the NZ population [45]. Also, a widely cited paper by Thomson et al., reported that the consumption of two Brazil nuts per day was as effective for improving selenium status as 100 μg of selenium in the form of a selenomethionine supplement in a group of New Zealanders [46]. This may explain why the fact that Brazil nuts contain substantial amounts of selenium is well known by health professionals in NZ. It would be interesting to compare this result with health professionals in different countries with different levels of selenium status and also to consider knowledge of the dangers, signs, and treatment of selenium toxicity.

Dietitians were more likely than GPs and/or practice nurses to recommend nuts because they are a good source of calories, unsaturated fatty acids, and fibre and because they promote satiety. An emphasis on energy, satiety, and certain nutrients such as fibre may reflect the nature of patients who dietitians are more likely to engage with, in comparison to patients of GPs and practice nurses, as well as dietitians’ specialist knowledge. Dietitians are more likely to have more in-depth consultations with individuals who want to gain or lose weight or who are looking to improve their overall diet quality.

Significantly more practice nurses promoted nuts as a good source of iron compared to both dietitians and GPs. Nuts provide around 0.5 to 2.8 mg of non-haem iron per 30 g [47] (which for 30 g of nuts equates to between 6% and 35% of the recommended dietary intakes (RDI) for adult men and post-menopausal women and between 3% and 16% for menstruating women) and are recommended to vegetarians and vegans as a source of iron [48]. This could be an area where patient education from health professionals might be beneficial, whereby nuts could be promoted to specific populations such as vegetarians and vegans.

GPs and practice nurses were twice as likely as dietitians to incorrectly believe that eating nuts could increase blood cholesterol concentrations. In addition, around 10% of GPs and practice nurses incorrectly believed that eating nuts could increase the risk of CVD, compared to less than 3% of dietitians. The perception of nut in regards to their effects on CVD and cholesterol-lowering differed significantly between dietitians and both GPs and practice nurses. Epidemiological evidence has consistently shown an inverse relationship between nut consumption and risk of CVD [4]. Further, numerous clinical trials have reported reductions in total and LDL-cholesterol with nut consumption [8]. This appears to be an area in which GPs and practice nurses could receive more education. This becomes even more apparent when analysing the reasons for advising patients to eat more nuts. Only 55%–60% of health professionals did so to reduce the risk of CVD and around half did so to lower blood cholesterol levels.

Weight gain has been reported as a barrier to regular nut consumption among the general public, who perceive nuts as ‘fattening’ [49]. Epidemiologic studies report that regular nut consumers are leaner than non-nut consumers [50,51,52,53]. Intervention studies where the main outcomes have been body weight have reported no weight gain or less weight gain than predicted based on energy content alone [54,55,56,57,58]. In addition, a meta-analysis of clinical trials reported that nut consumption was associated with non-significant decreases in body weight (0.47 kg), BMI (0.40 kg/m^2^), and waist circumference (1.25 cm) [34]. The lack of weight change with regular nut consumption may be explained by dietary compensation, inefficient energy absorption, and an increase in metabolic rate [27,28,34,59,60]. While collectively these data indicate that adding nuts to the diet does not result in weight gain, it should be noted that this research emphasises the inclusion of whole nuts into the diet, and this information should not be extrapolated to nuts in the form of snack bars and confectionery products. Ideally, nuts should replace less healthful snacks. In the current study, the majority of health professionals disagreed with the statement that eating nuts will cause people to gain weight. However, it is worthy to note that around one-fifth of GPs and practice nurses agreed that eating nuts caused weight gain. Therefore, there remains a substantial number of GPs and practice nurses who may inadvertently be adding to the confusion among the general public regarding the effects of regular nut consumption on body weight.

Reasons provided for advising patients to eat fewer nuts and nut butters appeared to be individualised to patients’ needs. For example, nuts being high in calories and fat and able to cause weight gain featured among the top three reasons. It is possible that health professionals would only advise overweight or obese patients to not eat nuts. Given the wealth of research showing that nut consumers tend to be leaner than non-nut consumers, health professionals may want to reconsider this advice.

One concern raised particularly among dietitians was dentition. Previous studies have compared the effects of consuming different forms of nuts, including ground, sliced, butter, and oil against whole nuts on blood lipids [61,62,63,64]. Collectively these studies found no significant differences in blood lipids between the different forms of nuts, providing alternatives to those who find whole nuts difficult to consume.

Concerns with nut allergies also featured as important. Nuts are one of the most common food allergens [65], and it is estimated that around 1% of the general population suffer from nut allergies [66,67]. Given that nut allergies can be severe and potentially life-threatening [68], health professionals should generally advise against nut consumption for those who have tested positive for a nut allergy.

Expense was among the top five reasons not to recommend nuts, especially among practice nurses. A recent cross-sectional survey showed that almost 50% of the participants agreed they would consume nuts if they were more affordable [15]. There are considerable differences in price between types of nuts. In New Zealand, peanuts are the least expensive at NZ$0.30 per 30 g serve (the amount recommended by the National Heart Foundation of NZ), followed by almonds and cashew nuts which cost less than NZ$0.80 per serve, with most other nuts, with the exception of pine nuts, costing less than NZ$1.50 per 30 g serve (these dollar amounts are approximately US$0.21, US$0.57, and US$1.07, respectively, at the present time). This cost is less than (for peanuts) or similar to (for other nuts) a serve of fruit, another healthy snack, and is comparable to less healthy snack foods, such as chocolate, cookies, crisps, and muesli bars. However, it should be noted that for low-income families, the cost of tree nuts could be prohibitive. Peanuts, which are most cost-effective, could be recommended to this group.

However, peanuts were in fact less likely to be recommended by health professionals compared to other nuts in this study. This was especially apparent among the GPs and practice nurses. When asked to rate the healthiness of peanuts compared to other nuts, almost half of the dietitians rated the healthiness of peanuts about the same as tree nuts, compared to only around one-quarter of GPs and practice nurses. The majority of GPs and practice nurses rated peanuts as ‘slightly less healthy’ than tree nuts. Peanuts, although a legume, have a similar nutrient composition to tree nuts, and their lower cost makes them a useful option for those for whom the cost of tree nuts might be a barrier. Given the results of this study, education targeted at health professionals on the health benefits of regular peanut consumption is warranted.

We examined whether any of the perceptions of nuts were predictors of whether health professionals would promote nut consumption. The only perception resulting in an increased likelihood of nut promotion across all three health professions in the adjusted models was that nuts reduce the risk of diabetes. There were a number of other predictors, which were specific to health professional-type. The identification of these predictors of nut promotion is useful as they can be incorporated into educational materials for health professionals in an attempt to increase the number of nut promoters and, consequently, nut consumers.

The strengths of the study include the careful development of the questionnaire to enhance face and content validity and several iterations of pre-testing to minimise misunderstandings on the part of respondents. The use of the Electoral Roll provided us with a representative sampling frame of New Zealanders, although there are some limitations with selecting participants based on the description of their occupation, which are discussed below. A further strength was the response rate, which was slightly higher than we had anticipated in the sample size calculations, achieved through the use of a rigorous mail survey using a modified version of Dillman’s Tailored Design Method with a mixed mode approach [42] to enhance the response rate.

There are also a number of limitations to bear in mind when interpreting the results of the present study. Firstly, although the response rate was 53%, which is comparable to other mail surveys conducted across Australasia [69,70], this still leaves the possibility that non-responders were systematically different from responders. If interest in and knowledge of nuts increased the likelihood of response, this would suggest that gaps in knowledge might be even larger than estimated. However, while estimated means, medians, and percentages might have been biased through respondents being more interested in the survey topic, there are no clear reasons why this would affect associations between responses. While studies investigating response biases in associations specific to health professionals are lacking, previous research has found no evidence for significant biases in associations involving health behaviours in the general public [71,72,73]. The cross-sectional nature of the study means that causal inferences cannot be drawn. However it seems more likely that a person’s training and experiences as a health professional would affect their perceptions and knowledge regarding nuts rather than the other way around, and the findings provide information with which to generate hypotheses for new studies in this area. The questionnaire was self-administered so it is not possible to determine whether the respondents fully understood the questions before answering; however careful development and pre-testing of the questionnaire was undertaken to address this as much as possible, and the respondents were from groups with high levels of education. It is possible that some potential participants described their occupation in ways that we did not consider and some descriptions were too general for us to include in the mail out (e.g., ‘doctor’ and ‘nurse’ would have been likely to capture mostly non-GP doctors and non-practice nurses). However, it is difficult to think of reasons why health professionals using different descriptions would differ from those sampled here. Similarly, some respondents may have retired recently and not have updated their occupation, although we did exclude descriptions indicating retirement from the selection process. The majority of respondents were female, which may limit the generalisability of the results to health professionals in general, although we adjusted for sex in regression models whenever possible to ensure that sex was not confounding associations.

## 5. Conclusions

In conclusion, we identified several areas in which health professionals, especially GPs and practice nurses, could receive further education on the health benefits of regular nut consumption, in particular the effects on blood cholesterol, CVD risk, and body weight. On the whole, dietitians appeared to have a greater understanding of the health benefits of nuts, which is likely to reflect their specialised nutrition training, but there is still room for improvement among responding dietitians. Improving the knowledge of the health benefits of nuts among health professionals is likely to improve the advice given to patients. This is important given the current low levels of nut consumption, despite the large body of literature reporting the health benefits of regular nut consumption.

## Figures and Tables

**Table 1:** Summary of the evidence for the health effects of nut consumption.

Disease/Risk Factor	Effect	Level of Evidence	Comments	Key References from Recent Meta-Analyses and Reviews
*Epidemiological studies*				
All-cause mortality	Decrease	Consistent evidence from few studies		[4,17,18,19,20,21]
CVD	Decrease	Consistent evidence from several studies		[4,17,18,19,20,21]
CHD/IHD	Decrease	Consistent evidence from several studies		[17,18,20]
Stroke	No change	Equivocal evidence from few studies	Some evidence for a reduction among females only	[17,18,19,20,22,23,24]
Hypertension	No change	Equivocal evidence from few studies		[24,25]
Diabetes	No change	Equivocal evidence from few studies	Some evidence for a weaker association after adjusting for BMI	[17,18,19,21,24,25,26]
Cancer (overall)	Decrease	Consistent evidence from few studies	Most evidence for colorectal, endometrial, and pancreatic cancers	[4,17,18,21,26]
Body weight	No change	Consistent evidence from several studies		[27,28]
Inflammatory markers	Decrease	Consistent evidence from few studies	Participants with higher nut intake also had healthier lifestyles	[29,30]
*Intervention studies*				
Total cholesterol	Decrease	Consistent evidence from several studies	Effects were more pronounced in participants with elevated cholesterol, BMI < 25, insulin sensitive	[8,31]
LDL-cholesterol	Decrease	Consistent evidence from several studies	Effects were more pronounced in participants with elevated cholesterol, BMI < 25, insulin sensitive	[8,31]
HDL-cholesterol	No change	Consistent evidence from several studies		[8,31]
Triglycerides	No change	Consistent evidence from several studies	Some evidence of a decrease among those with high baseline triglycerides	[8,31]
Systolic blood pressure	No change	Equivocal evidence from few studies	Some evidence of decreases seen among participants with hypertension	[31,32,33]
Diastolic blood pressure	No change	Equivocal evidence from few studies	Some evidence of decreases seen among participants with hypertension	[31,32,33]
Body weight	No change	Consistent evidence from several studies		[28,34]
Waist circumference	No change	Consistent evidence from several studies		[34,35]
Glycaemic control	No change	Equivocal evidence from few studies	Improvements may be more pronounced in people with type 2 diabetes	[32,35,36,37]
Antioxidants	Increase	Consistent evidence from several studies		[1,38]
Inflammatory markers	No change	Equivocal evidence from few studies	Improvements seen with PUFA-rich walnuts, and with further manipulation of the background diet	[32,38,39]
Endothelial function	No change	Equivocal evidence from few studies	Improvements seen with PUFA-rich walnuts, and with further manipulation of the background diet	[32,38]

Updated from Ros [3,40] and Tey et al. [9]. Abbreviations: BMI, body mass index; CHD, coronary heart disease; CVD, cardiovascular disease; IHD, ischaemic heart disease; HDL, high-density lipoprotein; LDL, low-density lipoprotein; PUFA, polyunsaturated fatty acids.

**Table 2:** Characteristics of study participants.

Demographic	All Health Professionals	Dietitians	General Practitioners	Practice Nurses	*p*-Value
*n*	759	318	292	149	
Female *n* (%)	617 (81%)	307 (97%)	167 (57%)	143 (96%)	<0.001
Age (years)	47.3 (11.1)	42.5 (12.0)	50.6 (8.4)	50.9 (10.3)	<0.001
Ethnicity *n* (%)					0.002
European	86 (649)	87 (277)	82 (240)	89 (132)	
Maori	4 (32)	6 (18)	3 (8)	4 (6)	
Pacific	1 (5)	1 (3)	<1 (1)	<1 (1)	
Asian	7 (55)	6 (18)	10 (30)	5 (7)	
MELAA	2 (12)	0 (0)	3 (10)	1 (2)	
Other	<1 (6)	<1 (2)	1 (3)	<1 (1)	
No. of years as a registered practitioner (median (IQR))	20.0 (18.0)	13.0 (20.0)	20.0 (15.0)	25.0 (17.5)	<0.001

All values are means (SD) unless otherwise specified. MELAA: Middle Eastern/Latin American/African. *p*-values from Chi-squared test (sex), one-way ANOVA (age), Fisher’s Exact test (ethnicity), and Kruskal-Wallis test (years registered).

**Table 3:** Responses from health professionals (%) regarding the perceptions and knowledge of nuts and nut butters.

**Statement**	**Dietitians (*n* = 318)**							**General Practitioners (*n* = 292)**						
	**Strongly Agree**	**Agree**	**Neither**	**Disagree**	**Strongly Disagree**	**Mean**	**Do not Know**	**Strongly Agree**	**Agree**	**Neither**	**Disagree**	**Strongly Disagree**	**Mean**	**Do Not Know**
They are low in energy/calories	0.6	0.6	4.4	40.3	54.1	4.4 ^a^	0	1.1	1.7	5.9	41.5	49.1	4.3 ^a^	0.7
They are high in protein	21.8	67.2	8.5	2.5	0	1	0 ^x^	20.1	65.1	8	5.2	0.4	2.0	1.4 ^x,y^
They are healthy	40.7	51.4	7.3	0.3	0.3	1.7 ^a^	0.0	24.5	55.2	15.3	4.2	0.7	2.0 ^b^	0.4
They are high in fat	38.6	57.0	2.5	1.0	1.0	1.7 ^a^	0.0	21.6	57.1	11.9	7.3	1.4	2.1 ^b^	0.7
They are filling	24.0	62.5	10.7	2.5	0.3	1.9	0 ^x^	18.8	66.3	9.0	3.5	0.7	1.9	1.7 ^y^
Some of them are high in selenium	48.3	46.4	1.9	1.0	0.3	1.5 ^a^	2.2 ^x^	21.5	50.4	8.3	0.4	0.7	2.0 ^b^	18.8 ^y^
They are low in vitamins & minerals	0.3	1.9	10.3	49.7	37.2	4.2 ^a^	0.6 ^x^	0.7	5.2	9.0	53.2	27.8	4.1 ^b^	4.2 ^y^
Eating them can increase people’s risk of cardiovascular disease	1.3	1.6	4.1	48.6	43.2	4.3 ^a^	1.3	0.4	9.4	21.5	43.8	20.5	3.7 ^b^	4.5
They are naturally high in salt/sodium	1.0	2.9	6.7	48.1	40.2	4.2 ^a^	0.6 ^x^	1.4	10.8	18.8	45.5	13.2	3.6 ^b^	10.4 ^y^
They are low in fibre	1.9	8.6	8.6	53.0	27.5	4.0 ^a^	0.3 ^x^	2.1	12.9	21.9	44.1	12.9	3.5 ^b^	6.3 ^y^
They are high in antioxidants	14.7	57.7	16.4	5.8	0.0	2.2 ^a^	5.5 ^x^	8.4	42.3	26.9	4.2	0.4	2.4 ^b^	17.8 ^y^
Eating them can increase people’s total blood cholesterol	0.7	9.5	10.1	50.5	27.1	3.9 ^a^	2.2 ^x^	1.4	20.1	22.5	36.7	12.5	3.4 ^b^	6.9 ^y^
Some of them are high in iron	5.5	35.4	21.2	23.2	4.2	2.8 ^a^	10.6 ^x^	3.5	33.3	25.4	11.5	1.4	2.7 ^a^	25.0 ^y^
Eating them can help lower people’s risk of diabetes	4.1	36.7	37.0	12.3	0.3	1.7	9.5 ^x^	5.5	32.2	29.4	15.6	1.0	2.3	16.3 ^y^
Eating them will cause people to gain weight	0.6	10.7	47.6	32.8	8.2	3.3	0.0	3.5	18.4	41.3	27.4	8.3	3.3	1.0
**Statement**	**Practice Nurses (*n* = 149)**													
	**Strongly Agree**	**Agree**	**Neither**	**Disagree**	**Strongly Disagree**	**Mean**	**Do not Know**	**Unadjusted *p*-Value †**	Adjusted *p*-Value †,*		**Unadjusted *p*-Value ‡**		Adjusted *p*-Value ‡,*	
They are low in energy/calories ^2^	3.4	4.8	7.5	56.5	27.2	3.9 ^b^	0.7	<0.001	<0.001		0.315 ^#^			
They are high in protein ^1^	24.2	65.8	6.7	1.3	0	1.8	2 ^y^	0.190	0.481		0.024 ^#^			
They are healthy ^1^	25.7	62.2	8.8	3.4	0.0	1.9 ^b^	0.0	<0.001	<0.001		0.578 ^#^			
They are high in fat ^1^	21.0	58.8	7.4	10.1	1.4	2.1 ^b^	1.4	<0.001	<0.001		0.112 ^#^			
They are filling ^1^	17.5	62.4	12.8	4.7	2.0	2.0	0.7 ^x,y^	0.066	0.058		0.042 ^#^			
Some of them are high in selenium ^1^	23.1	50.3	7.5	0.7	0.0	1.9 ^b^	18.4 ^y^	<0.001	<0.001		<0.001 ^∫^		<0.001	
They are low in vitamins & minerals ^2^	1.4	13.5	9.5	52.7	21.0	3.8 ^c^	2.0 ^x,y^	<0.001	<0.001		0.015 ^§^			
Eating them can increase people’s risk of cardiovascular disease ^2^	0.0	12.1	15.4	53.0	15.4	3.8 ^b^	4.0	<0.001	<0.001		0.071 ^∫^			
They are naturally high in salt/sodium ^2^	1.3	17.5	12.1	52.4	10.7	3.6 ^b^	6.0 ^y^	<0.001	<0.001		<0.001 ^∫^			
They are low in fibre ^2^	0.7	17.1	15.1	50.7	13.7	3.6 ^b^	2.7 ^x,y^	<0.001	<0.001		0.006 ^∫^			
They are high in antioxidants ^1^	8.7	53.0	16.1	6.7	0.7	2.3 ^a,b^	14.8 ^y^	0.007	0.028		<0.001 ^∫^		<0.001	
Eating them can increase people’s total blood cholesterol ^2^	2.7	19.5	15.4	48.3	8.1	3.4 ^b^	6.0 ^y^	<0.001	<0.001		0.026 ^∫^			
Some of them are high in iron ^3^	8.9	46.6	13.7	11.0	2.1	2.4 ^b^	17.8 ^y^	<0.001	<0.001		<0.001 ^∫^		<0.001	
Eating them can help lower people’s risk of diabetes ^4^	6.8	32.4	31.1	12.2	4.1	2.2	13.5 ^x,y^	0.986	0.963		0.047 ^∫^		0.050	
Eating them will cause people to gain weight ^2^	1.3	17.5	40.3	35.6	4.0	3.2	1.3	0.123	0.359		0.094 ^#^			

Responses scored: strongly agree = 1, agree = 2, neither = 3, disagree = 4, strongly disagree = 5; Neither: neither agree nor disagree. † *p*-value for differences between health professionals for mean responses (strongly agree to strongly disagree) calculated by linear regression. * Adjusted for age, sex, and ethnicity where there are sufficient responses; ‡ *p*-value for the difference between health professionals for answering ‘Do not know’; ^∫^ indicates logistic regression; § indicates Chi-square test; # indicates Fisher’s Exact test. When the overall *p-*values < 0.05, pairwise comparisons were performed. Values with different superscript letter indicate significant differences *p <* 0.05; Values with different superscript letters (a, b, c) are significantly different after adjustment for age, sex, and ethnicity. Values with different superscript letters (x, y, z) are significantly different in the unadjusted model for the response ‘Do not know’. Note that some statements are supported by current evidence and some are worded in contradiction to current evidence, ^1^ Statements that are supported by current evidence; ^2^ Statements that are contradicted by current evidence; ^3^ Some nuts such as pistachios, cashews, and almonds contain useful (>4 mg of non-haeme iron/100 g) amounts of iron, but their bioavailability and significance will rely on other dietary factors; ^4^ Statements where current evidence is uncertain.

**Table 4:** Reasons for advising patients to eat more nuts and nut butters among health professionals who promote nut consumption.

Reason	All Health Professionals (*n* = 519)	Dietitians	General Practitioners	Practice Nurses	Unadjusted *p*-Value	Adjusted *p*-Value *
		(*n* = 263)	(*n* = 162)	(*n* = 94)		
		% (*n*)				
They are a good source of protein	77.5 (402)	82.9 (218)	70.4 (114)	74.5 (70)	0.009	0.149
They are good for health/are nutritious	77.1 (400)	75.7 (199)	77.8 (126)	79.8 (75)	0.694	0.958
They are a good source of unsaturated fats	69.0 (358)	77.2 (203) ^a^	56.2 (91) ^b^	68.1 (64) ^a,b^	<0.001	0.001
They are a good source of energy/calories	65.9 (342)	74.5 (196) ^a^	55.6 (90) ^b^	59.6 (56) ^b^	<0.001	0.003
Eating them can help promote satiety	59.0 (306)	63.5 (167) ^a^	58.6 (95) ^a^	46.8 (44) ^b^	0.020	0.010
Some of them are a good source of selenium	58.4 (303)	55.9 (147)	61.7 (100)	59.6 (56)	0.480	0.340
Eating them can help decrease risk of CVD	58.8 (305)	61.2 (161) ^a^	56.2 (91) ^b^	56.4 (53) ^a,b^	0.517	0.038
They are a good source of vitamins and minerals	54.1 (281)	49.1 (129)	58.6 (95)	60.6 (57)	0.060	0.554
Eating them can help lower blood cholesterol	47.6 (247)	49.8 (131) ^a^	41.4 (67) ^b^	52.1 (49) ^a,b^	0.149	0.042
They are a good source of fibre	46.4 (241)	49.8 (131) ^a^	35.8 (58) ^b^	55.3 (52) ^a,b^	0.003	0.011
Eating them can help with weight management	38.5 (200)	37.6 (99)	39.5 (64)	39.4 (37)	0.914	0.915
They are a good source of antioxidants	35.5 (184)	33.1 (87)	34.0 (55)	44.7 (42)	0.119	0.403
Some of them are a good source of iron	25.6 (133)	17.9 (47) ^a^	29.0 (47) ^b^	41.5 (39) ^b^	<0.001	0.006

All values are presented as % (number); *p*-values calculated by logistic regression; * adjusted for age, sex, and ethnicity when there are sufficient responses. Values with different superscript letters are significantly different after adjustment for age, sex and ethnicity.

**Table 5:** Reasons for advising some patients to eat fewer nuts and nut butters.

Reason	All Health Professionals (*n* = 276)	Dietitians	General Practitioners	Practice Nurses	Unadjusted *p*-Value	Adjusted *p*-Value *
		(*n* = 131)	(*n* = 78)	(*n* = 67)		
		% (*n*)				
They are high in energy/calories	65.2 (180)	67.9 (89)	69.2 (54)	55.2 (37)	0.143 ^∫^	0.180 ^∫^
They are high in fat	39.1 (108)	31.3 (41) ^a^	46.2 (36) ^b^	46.3 (31) ^b^	0.042 ^∫^	0.148 ^∫^
Regular consumption of them can cause weight gain	35.1 (97)	28.2 (37)	41.0 (32)	41.8 (28)	0.075 ^∫^	0.404 ^∫^
There are concerns with nut allergy	21.7 (60)	18.3 (24)	25.6 (20)	23.9 (16)	0.413 ^∫^	
They are too expensive for patients	17.0 (47)	17.6 (23) ^a,b^	9.0 (7) ^a^	25.4 (17) ^b^	0.038 ^∫^	
Clients have more pressing concerns than nut consumption	14.5 (40)	9.9 (13)	16.7 (13)	20.9 (14)	0.102 ^∫^	
Dental issues make it inconvenient/uncomfortable for them	13.8 (38)	17.6 (23) ^a^	5.1 (4) ^b^	16.4 (11) ^a^	0.048 ^∫^	
Regular consumption of them can increase blood cholesterol	6.6 (18)	0 (0) ^a^	10.3 (8) ^b^	14.9 (10) ^b^	<0.001 ^§^	
They are naturally high in salt/sodium	6.5 (18)	1.5 (2) ^a^	11.5 (9) ^b^	10.5 (7) ^b^	0.006 ^§^	
Do not know enough about nuts & their benefits	5.8 (16)	0.8 (1) ^a^	6.4 (5) ^b^	14.9 (10) ^b^	<0.001 ^#^	
Regular consumption of them can increase the risk of CVD	4.7 (13)	0 (0) ^a^	7.7 (6) ^b^	10.5 (7) ^b^	<0.001 ^#^	
There is conflicting information & do not want to confuse patients	3.6 (10)	1.5 (2) ^a^	2.6 (2) ^a,b^	9.0 (6) ^b^	0.033 ^#^	
There is contraindication(s) with patients’ medication	0.7 (2)	0 (0)	1.3 (1)	1.5 (1)	0.275 ^#^	
They are unhealthy	0.7 (2)	0 (0)	1.3 (1)	1.5 (1)	0.275 ^#^	

All values are presented as % (number); * adjusted for age, sex, and ethnicity where there are sufficient responses. *p*-value for difference between health professionals, ^∫^ indicates logistic regression, ^§^ indicates Chi-square test, ^#^ indicates Fisher’s Exact test. Values with different superscript letters are significantly different in the unadjusted model.

**Table 6:** Associations between beliefs/perceptions and recommending nuts.

**Beliefs and Perceptions**	**Dietitians**			**General Practitioners**			**Practice Nurses**			
	**Unadjusted Odds Ratio**	**CI**	**Unadjusted *p*-Value**	**Unadjusted Odds Ratio**	**CI**	**Unadjusted *p*-Value**	**Unadjusted Odds Ratio**	**CI**	**Unadjusted *p*-Value**	**Unadjusted Overall *p*-Value for Interaction**
They are low in energy/calories ^2^	1.18	0.78, 1.78	0.428	1.01	0.75, 1.38	0.915	1.07	0.75, 1.54	0.710	0.849
They are high in protein ^1^	0.67	0.43, 1.05	0.079	0.83	0.60,1.15	0.260	0.85	0.48, 1.52	0.591	0.714
They are healthy ^1^	0.27 ^a^	0.16, 0.46	<0.001	0.38 ^a^	0.26, 0.54	<0.001	0.71 ^b^	0.44, 1.16	0.171	0.024
They are high in fat ^1^	0.79	0.52, 1.20	0.269	1.15	0.88, 1.52	0.303	0.90	0.62, 1.30	0.558	0.270
They are filling ^1^	0.73	0.49, 1.10	0.133	0.62	0.43, 0.88	0.008	1.14	0.75, 1.73	0.553	0.090
Some of them are high in selenium ^1^	0.64	0.41, 1.00	0.049	0.35	0.21, 0.56	<0.001	0.66	0.36, 1.23	0.195	0.129
They are low in vitamins & minerals ^2^	1.12	0.76, 1.67	0.564	1.89	1.36, 2.61	<0.001	1.22	0.87, 1.74	0.242	0.084
Eating them can increase people’s risk of cardiovascular disease ^2^	1.55	1.08, 2.22	0.018	1.69	1.28, 2.23	<0.001	1.52	1.02, 2.26	0.039	0.884
They are naturally high in salt/sodium ^2^	1.39	0.98, 1.96	0.066	1.89	1.14, 2.54	<0.001	1.60	1.11, 2.31	0.012	0.399
They are low in fibre ^2^	1.18	0.87, 1.59	0.283	1.32	1.02, 1.70	0.032	1.46	1.02, 2.09	0.040	0.659
They are high in antioxidants ^1^	0.75	0.50, 1.13	0.168	0.66	0.46, 0.94	0.023	0.81	0.51, 1.29	0.384	0.762
Eating them can increase people’s total blood cholesterol ^2^	1.46	1.08, 1.99	0.014	1.45	1.13, 1.85	0.003	1.29	0.91, 1.83	0.146	0.846
Some of them are high in iron ^3^	0.83	0.61, 1.13	0.234	0.57	0.41, 0.80	0.001	0.83	0.55, 1.23	0.345	0.210
Eating them can help lower people’s risk of diabetes ^4^	0.60 ^a,b^	0.40, 0.91	0.017	0.37 ^a^	0.26, 0.52	<0.001	0.69 ^b^	0.47, 1.01	0.055	0.041
Eating them will cause people to gain weight ^2^	1.21	0.84, 1.73	0.315	1.4	1.08, 1.80	0.010	1.58	1.04, 2.40	0.031	0.623
**Beliefs and Perceptions**	**Dietitians**			**General Practitioners**			**Practice Nurses**			
	Adjusted Odds Ratio *	**CI**	Adjusted *p*-Value *	Adjusted Odds Ratio *	**CI**	Adjusted *p*-Value *	Adjusted Odds Ratio *	**CI**	Adjusted *p*-Value *	Adjusted Overall *p*-Value for Interaction *
They are low in energy/calories ^2^	1.19	0.79, 1.80	0.406	0.97	0.71, 1.33	0.856	1.07	0.74, 1.54	0.721	0.737
They are high in protein ^1^	0.64	0.41, 1.00	0.051	0.87	0.62, 1.21	0.403	0.85	0.48, 1.51	0.578	0.539
They are healthy ^1^	0.27 ^a^	0.16, 0.45	<0.001	0.37 ^a^	0.25, 0.53	<0.001	0.74 ^b^	0.45, 1.21	0.231	0.016
They are high in fat ^1^	0.76	0.50, 1.16	0.210	1.22	0.92, 1.61	0.172	0.87	0.60, 1.27	0.473	0.137
They are filling ^1^	0.74	0.49, 1.12	0.151	0.64	0.44, 0.92	0.016	1.14	0.75, 1.74	0.543	0.115
Some of them are high in selenium ^1^	0.64	0.41, 1.01	0.057	0.35	0.21, 0.57	<0.001	0.65	0.35, 1.21	0.181	0.145
They are low in vitamins & minerals ^2^	1.19	0.79, 1.77	0.405	1.97	1.42, 2.75	<0.001	1.26	0.88, 1.79	0.210	0.087
Eating them can increase people’s risk of cardiovascular disease ^2^	1.55	1.08, 2.23	0.018	1.74	1.30, 2.32	<0.001	1.47	0.99, 2.19	0.059	0.774
They are naturally high in salt/sodium ^2^	1.39	0.98, 1.98	0.068	1.88	1.40, 2.54	<0.001	1.57	1.09, 2.27	0.017	0.420
They are low in fibre ^2^	1.19	0.88, 1.61	0.256	1.30	1.01, 1.68	0.043	1.46	1.01, 2.11	0.042	0.696
They are high in antioxidants ^1^	0.76	0.50, 1.15	0.191	0.62	0.43, 0.90	0.012	0.82	0.51, 1.31	0.406	0.622
Eating them can increase people’s total blood cholesterol ^2^	1.48	1.09, 2.02	0.013	1.43	1.11, 1.84	0.005	1.27	0.89, 1.79	0.186	0.790
Some of them are high in iron ^3^	0.85	0.62, 1.17	0.317	0.53	0.37, 0.75	<0.001	0.81	0.54, 1.22	0.317	0.106
Eating them can help lower people’s risk of diabetes ^4^	0.59 ^a,b^	0.39, 0.90	0.015	0.35 ^a^	0.25, 0.51	<0.001	0.65 ^b^	0.44, 0.97	0.034	0.050
Eating them will cause people to gain weight ^2^	1.20	0.83, 1.74	0.329	1.45	1.12, 1.89	0.006	1.52	1.00	0.052	0.640

Odds ratios (ORs) and *p*-values calculated from logistic regression and ORs reflect an increase of one point for the item (1 = strong agree, 5 = strongly disagree). * Adjusted for age, sex, and ethnicity. Values with different superscript letters are significantly different. Note that some statements are supported by current evidence and some are worded in contradiction to current evidence. ^1^ Statements that are supported by current evidence; ^2^ Statements that are contradicted by current evidence; ^3^ Some nuts such as pistachios, cashews, and almonds contain useful (>4 mg of non-haeme iron/100 g) amounts of iron, but their bioavailability and significance will rely on other dietary factors; ^4^ Statements where current evidence is uncertain.

**Table 7:** Perceived healthiness of peanuts in comparison to tree nuts as rated by health professionals.

	Dietitians (*n* = 314)		General Practitioners (*n* = 289)		Practice Nurses (*n* = 148)		Unadjusted *p*-Value	Adjusted *p*-Value *
	% (*n*)	Median	% (*n*)	Median	% (*n*)	Median		
5: Much more healthy	0.6 (2)	3: About the same ^a^	1.0 (3)	2: Slightly less healthy ^b^	0 (0)	2: Slightly less healthy ^b^	<0.001	<0.001
4: Slightly more healthy	1.6 (5)		1.4 (4)		1.4 (2)			
3: About the same	49.4 (155)		27.3 (79)		28.4 (42)			
2: Slightly less healthy	45.5 (143)		50.9 (147)		52.0 (77)			
1: Much less healthy	2.9 (9)		19.4 (56)		18.2 (27)			

*P*-value for differences between health professionals for median responses (much more healthy to much less healthy) calculated by quantile regression; * adjusted for age, sex, and ethnicity. Values with different superscript letters are significantly different.

## References

[1] Alasalvar C., Bolling B.W. (2015). Review of nut phytochemicals, fat-soluble bioactives, antioxidant components and health effects. Br. J. Nutr..

[2] Brufau G., Boatella J., Rafecas M. (2006). Nuts: Source of energy and macronutrients. Br. J. Nutr..

[3] Ros E. (2010). Health benefits of nut consumption. Nutrients.

[4] Grosso G., Yang J., Marventano S., Micek A., Galvano F., Kales S. (2015). Nut consumption and all-cause, cardiovascular, and cancer mortality risk: A systematic review and meta-analysis of epidemiologic studies. Am. J. Clin. Nutr..

[5] Hshieh T.T., Petrone A.B., Gaziano J.M., Djousse L. (2015). Nut consumption and risk of mortality in the Physicians’ Health Study. Am. J. Clin. Nutr..

[6] Kris-Etherton P.M., Yu-Poth S., Sabate J., Ratcliffe H.E., Zhao G., Etherton T.D. (1999). Nuts and their bioactive constituents: Effects on serum lipids and other factors that affect disease risk. Am. J. Clin. Nutr..

[7] Nash S.D., Nash D.T. (2008). Nuts as part of a healthy cardiovascular diet. Curr. Atheroscler. Rep..

[8] Sabate J., Oda K., Ros E. (2010). Nut consumption and blood lipid levels: A pooled analysis of 25 intervention trials. Arch. Intern. Med..

[9] Tey S., Brown R., Chisholm A. (2012). Nuts and Heart Health. National Heart Foundation of New Zealand Evidence-Based Position Statement on the Relationship of Nuts to Heart Health.

[10] US Food and Drug Administration Qualified Health Claims: Letter of Enforcement Discretion—Nuts and Coronary Heart Disease (Docket No. 02P-0505). http://www.fda.gov/Food/IngredientsPackagingLabeling/LabelingNutrition/ucm072926.htm.

[11] Brown R.C., Tey S.L., Gray A.R., Chisholm A., Smith C., Fleming E., Blakey C., Parnell W. (2014). Patterns and predictors of nut consumption: Results from the 2008/09 New Zealand Adult Nutrition Survey. Br. J. Nutr..

[12] Jenab M., Sabate J., Slimani N., Ferrari P., Mazuir M., Casagrande C., Deharveng G., Tyonneland A., Olsen A., Overvad K. (2006). Consumption and portion sizes of tree nuts, peanuts and seeds in the European Prospective Investigation into Cancer and Nutrition (EPIC) cohorts from 10 European countries. Br. J. Nutr..

[13] O’Neil C.E., Keast D.R., Nicklas T.A., Fulgoni V.L. (2012). Out-of-hand nut consumption is associated with improved nutrient intake and health risk markers in US children and adults: National Health and Nutrition Examination Survey 1999–2004. Nutr. Res..

[14] O’Neil C.E., Nicklas T.A., Fulgoni V.L. (2015). Tree nut consumption is associated with better nutrient adequacy and diet quality in adults: National Health and Nutrition Examination Survey 2005–2010. Nutrients.

[15] Pawlak R., Colby S., Herring J. (2009). Beliefs, benefits, barriers, attitude, intake and knowledge about peanuts and tree nuts among WIC participants in eastern North Carolina. Nutr. Res. Pract..

[16] Pawlak R., London H.A., Colby S.E., Wall-Bassett E., Sira N. (2012). Perception of nut intake among individuals with or at risk for heart disease and/or diabetes. J. Behav. Health.

[17] Aune D., Keum N., Giovannucci E., Fadnes L.T., Boffetta P., Greenwood D.C., Tonstad S., Vatten L.J., Riboli E., Norat T. (2016). Nut consumption and risk of cardiovascular disease, total cancer, all-cause and cause-specific mortality: A systematic review and dose-response meta-analysis of prospective studies. BMC Med..

[18] Bao Y., Han J., Hu F.B., Giovannucci E.L., Stampfer M.J., Willett W.C., Fuchs C.S. (2013). Association of nut consumption with total and cause-specific mortality. N. Engl. J. Med..

[19] Luo C., Zhang Y., Ding Y.S., Shan Z.L., Chen S.J., Yu M., Hu F.B., Liu L.G. (2014). Nut consumption and risk of type 2 diabetes, cardiovascular disease, and all-cause mortality: A systematic review and meta-analysis. Am. J. Clin. Nutr..

[20] Mayhew A.J., de Souza R.J., Meyre D., Anand S.S., Mente A. (2016). A systematic review and meta-analysis of nut consumption and incident risk of CVD and all-cause mortality. Br. J. Nutr..

[21] Van den Brandt P.A., Schouten L.J. (2015). Relationship of tree nut, peanut and peanut butter intake with total and cause-specific mortality: A cohort study and meta-analysis. Int. J. Epidemiol..

[22] Shao C., Tang H., Zhao W., He J. (2016). Nut intake and stroke risk: A dose-response meta-analysis of prospective cohort studies. Sci. Rep..

[23] Shi Z.Q., Tang J.J., Wu H., Xie C.Y., He Z.Z. (2014). Consumption of nuts and legumes and risk of stroke: A meta-analysis of prospective cohort studies. Nutr. Metab. Cardiovasc. Dis..

[24] Zhou D.H., Yu H.B., He F., Reilly K.H., Zliang J.L., Li S.S., Zhang T., Wang B.Z., Ding Y.L., Xi B. (2014). Nut consumption in relation to cardiovascular disease risk and type 2 diabetes: A systematic review and meta-analysis of prospective studies. Am. J. Clin. Nutr..

[25] Guo K., Zhou Z., Jiang Y., Li W., Li Y. (2015). Meta-analysis of prospective studies on the effects of nut consumption on hypertension and type 2 diabetes mellitus mamm. J. Diabetes.

[26] Wu L., Wang Z., Zhu J., Murad A.L., Prokop L.J., Murad M.H. (2015). Nut consumption and risk of cancer and type 2 diabetes: A systematic review and meta-analysis. Nutr. Rev..

[27] Jackson C.L., Hu F.B. (2014). Long-term associations of nut consumption with body weight and obesity. Am. J. Clin. Nutr..

[28] Tan S.Y., Dhillon J., Mattes R.D. (2014). A review of the effects of nuts on appetite, food intake, metabolism, and body weight. Am. J. Clin. Nutr..

[29] Casas-Agustench P., Bullo M., Salas-Salvado J. (2010). Nuts, inflammation and insulin resistance. Asia Pac. J. Clin. Nutr..

[30] Yu Z., Malik V.S., Keum N., Hu F.B., Giovannucci E.L., Stampfer M.J., Willett W.C., Fuchs C.S., Bao Y. (2016). Associations between nut consumption and inflammatory biomarkers. Am. J. Clin. Nutr..

[31] Del Gobbo L.C., Falk M.C., Feldman R., Lewis K., Mozaffarian D. (2015). Effects of tree nuts on blood lipids, apolipoproteins, and blood pressure: Systematic review, meta-analysis, and dose-response of 61 controlled intervention trials. Am. J. Clin. Nutr..

[32] Barbour J.A., Howe P.R., Buckley J.D., Bryan J., Coates A.M. (2014). Nut consumption for vascular health and cognitive function. Nutr. Res. Rev..

[33] Mohammadifard N., Salehi-Abarghouei A., Salas-Salvado J., Guasch-Ferre M., Humphries K., Sarrafzadegan N. (2015). The effect of tree nut, peanut, and soy nut consumption on blood pressure: A systematic review and meta-analysis of randomized controlled clinical trials. Am. J. Clin. Nutr..

[34] Flores-Mateo G., Rojas-Rueda D., Basora J., Ros E., Salas-Salvadó J. (2013). Nut intake and adiposity: Meta-analysis of clinical trials. Am. J. Clin. Nutr..

[35] Blanco Mejia S., Kendall C.W., Viguiliouk E., Augustin L.S., Ha V., Cozma A.I., Mirrahimi A., Maroleanu A., Chiavaroli L., Leiter L.A. (2014). Effect of tree nuts on metabolic syndrome criteria: A systematic review and meta-analysis of randomised controlled trials. BMJ Open.

[36] Salas-Salvado J., Guasch-Ferre M., Bullo M., Sabate J. (2014). Nuts in the prevention and treatment of metabolic syndrome. Am. J. Clin. Nutr..

[37] Viguiliouk E., Kendall C.W., Blanco Mejia S., Cozma A.I., Ha V., Mirrahimi A., Jayalath V.H., Augustin L.S., Chiavaroli L., Leiter L.A. (2014). Effect of tree nuts on glycemic control in diabetes: A systematic review and meta-analysis of randomized controlled dietary trials. PLoS ONE.

[38] Banel D.K., Hu F.B. (2009). Effects of walnut consumption on blood lipids and other cardiovascular risk factors: A meta-analysis and systematic review. Am. J. Clin. Nutr..

[39] Mazidi M., Rezaie P., Ferns G.A., Gao H.-K. (2016). Impact of different types of tree nut, peanut, and soy nut consumption on serum C-reactive protein (CRP): A systematic review and meta-analysis of randomized controlled clinical trials. Medicine.

[40] Ros E. (2015). Nuts and CVD. Br. J. Nutr..

[41] Electoral Commission. http://www.elections.org.nz/research-statistics/enrolment-statistics-electorate.

[42] Dillman D. (2011). Mail and Internet Surveys: The Tailored Design Method—2007 Update with New Internet, Visual, and Mixed-Mode Guide.

[43] Statistics New Zealand (2005). The Statistical Standard for Ethnicity 2005.

[44] Peduzzi P., Concato J., Kemper E., Holford T.R., Feinstein A.R. (1996). A simulation study of the number of events per variable in logistic regression analysis. J. Clin. Epidemiol..

[45] Thomson C.D. (2004). Selenium and iodine intakes and status in New Zealand and Australia. Br. J. Nutr..

[46] Thomson C.D., Chisholm A., McLachlan S.K., Campbell J.M. (2008). Brazil nuts: An effective way to improve selenium status. Am. J. Clin. Nutr..

[47] Lesperance L. (2009). The Concise New Zealand Food Composition Tables.

[48] Messina V., Melina V., Mangels A.R. (2003). A new food guide for North American vegetarians. J. Am. Diet. Assoc..

[49] Webb Y., Dear W. (1996). Slimmers’ knowledge, beliefs and practices about fat, cholesterol and egg intake. Food Aust..

[50] Bes-Rastrollo M., Sabate J., Gomez-Gracia E., Alonso A., Martinez J.A., Martinez-Gonzalez M.A. (2007). Nut consumption and weight gain in a Mediterranean cohort: The SUN Study. Obesity.

[51] Bes-Rastrollo M., Wedick N.M., Martinez-Gonzalez M.A., Li T.Y., Sampson L., Hu F.B. (2009). Prospective study of nut consumption, long-term weight change, and obesity risk in women. Am. J. Clin. Nutr..

[52] Martinez-Gonzalez M.A., Bes-Rastrollo M. (2011). Nut consumption, weight gain and obesity: Epidemiological evidence. Nutr. Metab. Cardiovasc. Dis..

[53] Mozaffarian D., Hao T., Rimm E.B., Willett W.C., Hu F.B. (2011). Changes in diet and lifestyle and long-term weight gain in women and men. N. Engl. J. Med..

[54] Alper C.M., Mattes R.D. (2002). Effects of chronic peanut consumption on energy balance and hedonics. Int. J. Obes. Relat. Metab. Disord..

[55] Fraser G.E., Bennett H.W., Jaceldo K.B., Sabate J. (2002). Effect on body weight of a free 76 kilojoule (320 calorie) daily supplement of almonds for six months. J. Am. Coll. Nutr..

[56] Hollis J., Mattes R. (2007). Effect of chronic consumption of almonds on body weight in healthy humans. Br. J. Nutr..

[57] Sabate J., Cordero-MacIntyre Z., Siapco G., Torabian S., Haddad E. (2005). Does regular walnut consumption lead to weight gain?. Br. J. Nutr..

[58] Tey S.L., Brown R., Gray A., Chisholm A., Delahunty C. (2011). Nuts improve diet quality compared to other energy-dense snacks while maintaining body weight. J. Nutr. Metab..

[59] Grundy M., Grassby T., Mandalari G., Waldron K., Butterworth P.J., Berry S., Ellis P. (2015). Effect of mastication on lipid bioaccessibility of almonds in a randomized human study and its implications for digestion kinetics, metabolizable energy, and postprandial lipemia. Am. J. Clin. Nutr..

[60] Mattes R.D., Dreher M.L. (2010). Nuts and healthy body weight maintenance mechanisms. Asia Pac. J. Clin. Nutr..

[61] Hyson D.A., Schneeman B.O., Davis P.A. (2002). Almonds and almond oil have similar effects on plasma lipids and LDL oxidation in healthy men and women. J. Nutr..

[62] McKiernan F., Lokko P., Kuevi A., Sales R.L., Costa N.M., Bressan J., Alfenas R.C., Mattes R.D. (2010). Effects of peanut processing on body weight and fasting plasma lipids. Br. J. Nutr..

[63] Spiller G.A., Miller A., Olivera K., Reynolds J., Miller B., Morse S.J., Dewell A., Farquhar J.W. (2003). Effects of plant-based diets high in raw or roasted almonds, or roasted almond butter on serum lipoproteins in humans. J. Am. Coll. Nutr..

[64] Tey S.L., Brown R.C., Chisholm A.W., Delahunty C.M., Gray A.R., Williams S.M. (2011). Effects of different forms of hazelnuts on blood lipids and α-tocopherol concentrations in mildly hypercholesterolemic individuals. Eur. J. Clin. Nutr..

[65] Davis P.A., Jenab M., Vanden Heuvel J.P., Furlong T., Taylor S. (2008). Tree nut and peanut consumption in relation to chronic and metabolic diseases including allergy. J. Nutr..

[66] Crespo J.F., James J.M., Fernandez-Rodriguez C., Rodriguez J. (2006). Food allergy: Nuts and tree nuts. Br. J. Nutr..

[67] Fitzharris P. (2001). Peanut allergy. Prescriber Update.

[68] Sinclair J. (2013). Fatal food allergy and opportunities for risk minimisation. NZ Med. J..

[69] Lee C., Dobson A.J., Brown W.J., Bryson L., Byles J., Warner-Smith P., Young A.F. (2005). Cohort Profile: The Australian Longitudinal Study on Women’s Health. Int. J. Epidemiol..

[70] Timperio A., Cameron-Smith D., Burns C., Salmon J., Crawford D. (2000). Physical activity beliefs and behaviours among adults attempting weight control. Int. J. Obes. Relat. Metab. Disord..

[71] Martikainen P., Laaksonen M., Piha K., Lallukka T. (2007). Does survey non-response bias the association between occupational social class and health?. Scand. J. Public Health.

[72] Mealing N.M., Banks E., Jorm L.R., Steel D.G., Clements M.S., Rogers K.D. (2010). Investigation of relative risk estimates from studies of the same population with contrasting response rates and designs. BMC Med. Res. Methodol..

[73] Van Loon A.J., Tijhuis M., Picavet H.S., Surtees P.G., Ormel J. (2003). Survey non-response in the Netherlands: Effects on prevalence estimates and associations. Ann. Epidemiol..

